# Development and Characterization of Active Native and Cross-Linked Pearl Millet Starch-Based Film Loaded with Fenugreek Oil

**DOI:** 10.3390/foods10123097

**Published:** 2021-12-14

**Authors:** Sanju Bala Dhull, Sneh Punia Bangar, Ranjan Deswal, Preeti Dhandhi, Manish Kumar, Monica Trif, Alexandru Rusu

**Affiliations:** 1 Department of Food Science and Technology, Chaudhary Devi Lal University, Sirsa 125055, Haryana, India; sanjudhull@gmail.com (S.B.D.); ranjandeswal7@gmail.com (R.D.); preetidhandi@gmail.com (P.D.); 2 Department of Food, Nutrition and Packaging Sciences, Clemson University, Clemson, SC 29631, USA; 3 Department of Food Technology, GJUST HISAR, Hisar 125001, Haryana, India; manishgju@gjust.org; 4 Centre for Innovative Process Engineering (CENTIV) GmbH, 28857 Syke, Germany; monica_trif@hotmail.com; 5 Life Science Institute, University of Agricultural Sciences and Veterinary Medicine Cluj-Napoca, 400372 Cluj-Napoca, Romania

**Keywords:** pearl millet starch, fenugreek oil, biocomposite films, properties

## Abstract

In this study, cross-linked pearl millet starch and fenugreek oil was used to develop active starch edible films to overcome the limitations of native starch and to substitute artificial preservatives with natural one. The starch was cross-linked at three levels (1%, 3% and 5%) using sodium trimetaphosphate (STMP), and physicochemical properties were studied. Moreover, a comparative study was conducted among four samples of films prepared using native starch, cross-linked starch, and native and cross-linked starch loaded with fenugreek oil for physical, thermal, mechanical, morphological, and antibacterial properties. The solubility, swelling, and amylose content of native and modified starch varied from 11.25–12.75%, 12.91–15.10 g/g, and 8.97–16.55%, respectively. The values of these parameters were reduced as the concentration of STMP increased. Cross-linked starch films showed lower moisture, solubility, water vapor permeability(WVP), and elongation at break (EB) values while having higher thickness, opacity, thermal, and tensile strength values. The microscopic images of cross-linked starch films showed smooth surfaces and the absence of ridges, pores, and cracks. The films loaded with fenugreek oil showed different results; the moisture content, water solubility, and tensile strength were decreased while thickness, opacity, WVP, and EB were increased. The onset temperature and peak temperature were lower, while enthalpy of gelatinization was increased to a greater extent than films without oil. The addition of fenugreek oil to films showed a good inhibition area of 40.22% for native+oil films and 41.53% for cross-linked+oil films % against *Escherichia coli.* This study confirmed the successful utilization of fenugreek oil as a very effective antimicrobial agent in preparing edible films.

## 1. Introduction

There is a rising concern for using petroleum-based plastics for food packaging as they are non-biodegradable, thus leaving long-lasting environmental problems. The degradation of these polymers on earth typically leads to the toxicity of the environment. Their waste in water bodies may have new or unintended harmful environmental consequences [1]. The emissions from burning these plastics result in the release of greenhouse gases, which results in environmental issues such as global warming, which affects all forms of life [2]. Polysaccharides derived from renewable sources such as gums (seaweed-based gums, e.g., alginate, carrageenan, etc.; plant-origin gums, e.g., Arabic gum, karaya gum, mastic gum, etc.; and microbial gums, e.g.,gellan, xanthan, pullulan, bacterial cellulose, etc.) and starches, are attracting interest for film/coating applications in different industries. Starches are considered a very efficient base for decomposable plastic films due to their low raw material cost and easy availability. They can easily be processed with available plastic-processing equipment [3,4]. Nowadays, many efforts have been made to find alternatives and ecological substitutes that can replace plastic packaging, and as a solution, starch films may be used [5].

The availability and ease of starch extraction make pearl millet grain a good source, as about 59–80% of its overall components is starch [6]. The starch granules of pearl millet are spherical and polygonal in shape, varying from small to large. It also has deep indents and pores on the surface, facilitating its modification and digestibility [7,8]. Compared with cereal starches, pearl millet starch has shown better physicochemical properties, which proves it as a suitable and economical replacement useful for starch-based industries. However, due to several limiting characteristics such as poor viscosity, solubility, a higher degree of retrogradation, and low shear stability, native pearl millet starch has limited applications in different food products. Therefore, different modifications, including chemical, physical, enzymatic or dual, are suggested to improve the functionalities of pearl millet starch and expand its uses. After different modifications, the literature has confirmed different functional, physicochemical, morphological, thermal, pasting, and rheological properties of pearl millet starch [9]. Both native and modified pearl millet starch has many applications such as edible films, coatings, pharma, medical uses, nanoparticle development, food bulking, thickening and gelling agents, and fat replacer colloid stabilizer, etc. [10].Films made with starch are tasteless, odorless, flexible, of moderate strength, transparent, and moderately permeable to CO_2_, O_2,_ and moisture [11]. Using native starch to prepare edible films brings undesirable properties such as higher water vapor permeability, unattractive appearance, lesser stability during thermal and mechanical operations [10,12]. Starch modification can overcome these undesirable properties by using chemical and physical agents to improve starches’ applicability [12]. A simultaneous utilization in filmsofstarchin combination with hydrocolloids (such as guar gum, xanthan gum) or pectin (which for some applications is too brittle) can modify the film’s mechanical resistance [13,14].

Modification can be achieved by various means such as chemical, physical, genetic, and enzymatic [10]. These modifications will provide much-needed functionality to starch films and open a new window for starch usage in various food sector applications [15,16]. Chemical modification is achieved by using different chemical reagents such as alkylene oxide, vinyl acetate, acetic anhydride, phosphoryl chloride (POCl_3_), epichlorohydrin, and sodium trimetaphosphate (STMP) [10]. Among all types of modifications, chemical modification by cross-linking provides better results than others. STMP has have been an efficient cross-linking agent due to its nontoxic nature and slower penetration rate. Starch-modified biodegradable films with STMP are shown to have higher thermal stability, tensile strength, opacity, and decreased water vapor permeability [17].

Synthetic preservatives are mostly used today compared to natural ones due to their easy availability. Researchers have reported that the use of artificial preservatives such as nitrates, sorbates, parabens, formaldehyde, benzoates, sulfites, butylated hydroxytoluene (BHT), butylated hydroxyanisole (BHA), and several others leads to serious health hazards such as asthma, hyperactivity, hypersensitivity, allergy, neurological damage, and cancer [18,19,20]. These harmful impacts of synthetic preservatives have created possibilities for researchers to investigate new advances in active packaging systems containing natural bioactive compounds such as essential oils [21]. These active packaging systems provide microbial safety for consumers and extend the shelf life of packaged food and replace synthetic preservatives [22,23]. This led researchers to investigate plants as a source of new antimicrobial agents [24]. *Trigonella foenum-graecum* is an annual legume commonly known as Fenugreek, which belongs to the Fabaceae family. It has been proven that fenugreek oil’s components such as phenolics, alkaloids, anthracene glycosides, flavonoids, saponins, tannin, volatile oils, and phenolics make it an astonishing antimicrobial agent [25].

Fenugreek oil (*T. foenum-graecum*) has potential preservation effects against various microbe strains that cause food-borne illness, and therefore, can be substituted for chemical preservatives. Alwhibi et al. [24] reported the effectiveness of fenugreek oil against both gram-positive and Gram-negative bacterial isolates, including *E. coli*, *Pseudomonas aeruginosa*, *Klebsiella pneumonia, Staphylococcus aureus,* and *Salmonella Typhi*. In the past, a limited study was conducted on the effect of cross-linking on the properties of the starch film. Little data is available on incorporating fenugreek seed oil in edible film preparations and its effect on different functional and microbiological properties. Thus, the present study explored the preparation of edible films using STMP cross-linked pearl millet starch and comparatively studied its different properties with native starch film. Further, the investigation also explored the effect of the incorporation of fenugreek seed oil on different properties of the obtained film.

## 2. Materials and Methods

### 2.1. Material

Pearl millet grains were procured from Chaudhary Charan Singh Haryana Agricultural University, Hisar, India. All chemicals used were of analytical grade.

### 2.2. Starch Extraction

Starch was extracted from pearl millet grain by following the methods described in previous studies [26]. Whole grains (500g), with a moisture content of 20%, were steeped in 1.25 L of distilled water containing 0.1% sodium metabisulfite (≥97%, Sigma-Aldrich, St. Louis, MO, USA) at 50°C for 18–20 h. After the grains had softened, they were milled to a slurry. Then, the slurry was passed through different sieves of mesh sizes 45, 75, 100, 150, and 250 to separate protein and other fibrous matter. After sieving, the starch protein slurry was kept for 4–5 h in a refrigerator (REMI, RLR-300, Mumbai, MH, India) at a temperature of 4 °C. Then, the supernatant was removed, and the settled starch layer was resuspended in chilled water. The slurry was then centrifuged at 3000 rpm for 10 min using a centrifuge (Eltek, TC 8100F, Mumbai, MH, India). Then, the upper brownish layer was scraped, and the white layer was again suspended in water. This procedure was repeated 3–4 times to obtain pure starch. After that, the starch was allowed to dry in a hot air oven (NSW-143, New Delhi, India) at 45 °C for 18 h.

### 2.3. Modification of Starch

The cross-linked pearl millet starch was prepared following the method Woo and Seib [27] explained. A 100 g of starch was dispersed in distilled water (250 mL). Then, sodium trimetaphosphate (STMP, ≥95%, Sigma-Aldrich, St. Louis, MO, USA) (1.0%, 3.0%, and 5.0%) was added to the slurry, and we adjusted the pH of the mixture to 10.5 by using 5% NaOH (≥98%, Sigma-Aldrich, St. Louis, MO, USA) solution. The slurries were then maintained at 45 °C/1 h with continuous stirring. After that, using 2% HCl(≥37%, Sigma-Aldrich, St. Louis, MO, USA) solution, we adjusted pH to 5.5 to stop the reaction. Finally, washing of slurry with distilled water was performed till it was neutralized, followed by centrifugation (Eltek, TC 8100F, Mumbai, MH, India) at 3000 rpm for 10 min, and drying of the starch at 45 °C for 24 h in a hot air oven (NSW-143, New Delhi, India)

### 2.4. Fenugreek Oil Extraction

Fenugreek oil (*Trigonella foenumgraecum*) was extracted by following the method of Akbari et al. [28] with slight modifications. Fenugreek oil was extracted by using the soxhlet apparatus. The solvent n-hexane (≥95%, Sigma-Aldrich, St. Louis, MO, USA) was run through the fenugreek crushed seeds at a temperature of 65–70 °C for 4 h. The obtained mixture was then filtered through a No. 1 filter paper. The extract was then transferred to a glass beaker, and the solvent was evaporated using a hot air oven (NSW-143, New Delhi, India) at 47 °C for 24 h. Finally, the extracted oil was stored at 4 °C(REMI, RLR-300, Vasai, MH, India) to avoid degradation for further analysis [29].

### 2.5. Amylose Content

The amylose content of pearl millet starch was measured as defined by Williams et al. [30]. A total of 20g of pearl millet starch was dispersed in KOH (Central Drug House (P) Ltd., New Delhi, India) solution (0.5N) and thoroughly mixed using a magnetic stirrer (SPINIT, Tarson, Kolkata, India). Further, the solution was transferred to a 100 mL volumetric flask and diluted using distilled water up to the mark. An aliquot of 10 mL test starch solution was transferred into a 50 mL volumetric flask, and we added 5 mL of 0.1 N HCl followed by 0.5 mL of iodine reagent. The volume was made to 50 mL using distilled water, and absorbance was recorded at 625 nm in a UV–VIS spectrophotometer (Systronics-2203, Ahmadabad, GJ, India). The amylose content was calculated using the following equation [31]:

Amylose content = 85.24X − 13.19(1)

where X = absorbance

### 2.6. Swelling Power (SP) and Solubility

The SP and solubility of pearl millet starch were calculated by adopting the method of Leach et al. [32]. Then, 1 g of pearl millet starch was mixed with 99 mLdistilled water followed by heating at 90 °C for 1 h. Next, the samples were cooled quickly in an ice-water bath for 1 min, kept at 25 °C for 5 min, and centrifuged (Eltek, TC 8100F, Mumbai, MH, India) in preweighed centrifuge tubes at 3000 rpm for 20 min. The supernatant was drained into a preweighed Petri plate, and the sediment was weighed separately. The supernatant was dried in a hot air oven (NSW-143, New Delhi, India at 100 °C for 24 h and then cooled to room temperature in a desiccator before reweighing.

Calculations [33]:

(2) $$\mathrm{Swelling}\;\mathrm{power}\;\left(g/g\right)=\frac{\mathrm{Weight}\;\mathrm{of}\;\mathrm{sediment}}{\mathrm{Initial}\;\mathrm{weight}\;\mathrm{of}\;\mathrm{the}\;\mathrm{dry}\;\mathrm{starch}}$$

Calculations [34]

Solubility (%) = (Weight of dried supernatant)/(Initial weight of the dry starch) × 100 (3)

### 2.7. Paste Clarity

Light transmittance (%) of pastes of pearl millet starch was calculated following the method of Perera and Hoover [35]. An aqueous suspension of 1% pearl millet starch(dry basis) was heated to 90°C for 30 min in a boiling water bath (Brookfield, TC-202, Harlow, UK) with constant stirring. Further, the suspension was allowed to cool to room temperature and stored at 4 °C for 5 days. The transmittance was taken at 640 nm using a UV–VIS Spectrophotometer (Systronics-2203, Ahmadabad, GJ, India) for six days, using water as blank.

### 2.8. Preparations of Cross-Linked Pearl Millet Starch Films

The film was formed by following the procedure of Sharma et al. [17] with slight modifications. For film formulation, 4 g of starch was dispersed in 100 mL of distilled water with constant stirring at 200 rpm. Then, 2 g of glycerol was added to the solution to mix properly for 20 min on the stirrer. Then, the starch was gelatinized at 80 °C for 20 min in a water bath (Brookfield, TC-202, Harlow, UK) with periodic stirring so that lumps of gelatinized starch not formed. After gelatinization, the solution was allowed to cool at room temperature. The resulting solution was cast on a Teflon-coated tray, using a muslin cloth for filtration. The film solution was allowed to dry for 22–24 h at 45 °C in a conventional hot air oven (NSW-143, New Delhi, India). After drying, the films were peeled off and stored at 25 °C and relative humidity of 50%for 48 h for further analysis. The preparation of oil-incorporated films and Souza et al. [22] procedure was followed with slight modifications. The previous steps were followed while adding 0.80 gm fenugreek oil and 0.100 gm of emulsifier during the stirring step [36].

### 2.9. Moisture Content

The moisture content of the films was measured by the oven-drying method. Films were kept at 100 °C in a hot air oven (NSW-143, New Delhi, India) for 24 h. Samples were evaluated at least in triplicate, and results were calculated as (%).

### 2.10. Film Thickness

The thickness of the films was calculated using a digital micrometer with an accuracy and precision of ±0.001. Thickness was measured at ten different locations for individual film, and the mean value was calculated.

### 2.11. Opacity of Films

Film opacity was calculated by the method explained by Ramos et al. [37]. Rectangular strips (2 mm × 7 mm) of preconditioned films were placed onto the surface of a cuvette. Taking the empty cuvette as a reference, the absorbance of film samples were reported at 600 nm using a UV–visible spectrophotometer (Systronics-2203, Ahmadabad, GJ, India) and calculated by using the following equation [17]:

(4) $$\mathrm{Opacity}\;=\frac{A600}{X}$$

where A600 was the absorbance at 600 nm, and “X” was the film thickness (mm).

### 2.12. Water Solubility of Films

The water solubility of film samples was evaluated following Romero-Bastida et al. [38]. Strips of film samples 2 cm × 2 cm were cut out and dried in a hot air oven (NSW-143, New Delhi, India) for 24 h at 100 °C. We weighed the dried film sample, immersed it in 100 mLconical flasks containing 80 mL of distilled water, and shook it slightly, periodically, for one hour. We carefully separated the nonsolubilized film, then dried it at 100 °C for 24 h. The obtained matter was weighed, and % solubility was calculated as [38]:

(5) $$\%\;\mathrm{Solubility}=\frac{\mathrm{Initial}\;\mathrm{dry}\;\mathrm{weight}\;-\;\mathrm{Final}\;\mathrm{dry}\;\mathrm{weight}}{\mathrm{Initial}\;\mathrm{dry}\;\mathrm{weight}}\times 100\;$$

### 2.13. Water Vapor Permeability (WVP)

WVP of films was determined by the gravimetric modified cup method, based on ASTM E96/E96M [39]. We tookroughly 50 g of anhydrous CaCl_2_ (Thermo Fisher Scientific India Pvt. Ltd., Mumbai, Maharashtra, India) as a desiccant in the cup and sealed the mouth with film using a sealant. A temperature of 30 °C and RH of 75% was maintained using a saturated solution of NaCl inside the permeation cell. The weight-gain measurements were recorded at 3 h for 48 h. Measurements were taken in triplicate, and their mean value was evaluated. After measuring the permeation, WVTR was calculated using the following equation [40]:

(6) $$\mathrm{WVTR}\;=\frac{G}{\mathrm{tA}}$$

where G is weight change (g), t is time (s) and A was test area (cup mouth area, m^2^).

WVP (g m Pa^−1^ s^−1^ m^−2^) was calculated using the following equation [40]:

(7) $$\mathrm{WVP}\;=\frac{\mathrm{WVTR}}{S\left(R1-R2\right)}\times d$$

where S is the saturation vapor pressure of water (Pa) at the test temperature (30 °C), R1 is the RH (%) in a humidity chamber, R2 is the RH (%) in the cup, and d is the film thickness (m).

### 2.14. Differential Scanning Calorimetry (DSC)

Differential scanning calorimetry of film samples was performed on DSC (Discovery-25, TA Instruments, New Castle, Delaware, USA). The film sample (8–10 mg) was cut into small cuts, weighed in an aluminum pan, and sealed hermetically. An empty pan was used as a reference. The samples were analyzed over a temperature range of 20 to 200 °C with a heating rate of 10 °C/min. The glass transition temperature (T_g_) and enthalpy of melting (ΔH_m_) were determined from the resulting thermograms.

### 2.15. Tensile Properties of Films

The tensile properties of the films were determined by the method provided by ASTM [41] using a texture analyzer (TA-XT2i, Stable Micro Systems, Godalming, Surrey, UK). A probe A/TG Tensile Grip was used. For the measurement, films strip sizes of 10 mm × 50 mm were fixed between the separation grips of the machine. Initial grip separation and crosshead speeds were 20 mm and 1 mm/s, respectively. Both tensile strength (TS) and elongation at break (EB) were calculated by using the texture analyzer. Tensile strength was calculated by using the following equation [42]:

(8) $$\mathrm{Tensile}\;\mathrm{Strength}\;\left(\mathrm{TS}\right)=\frac{\mathrm{Force}\left(N\right)}{\;\mathrm{Width}\;\left(\mathrm{mm}\right)\times \mathrm{thickness}\;\left(\mathrm{mm}\right)}$$

EB is the percentage change calculated by dividing the film elongation at the moment of rupture by the initial gauge length of the film, multiplied by 100. Measurements were carried out in triplicate, and their mean value was taken.

### 2.16. Scanning Electron Microscopy (SEM)

The surface of dried films was imaged by scanning electron microscope (JSM-6100, Joel, Scotia, NY, USA). The film samples were mounted on specimen holder stubs with double-sided carbon adhesive tape; next, a thin layer of gold was sputtered. SEM images were taken at an accelerating voltage of 10 kV and magnifications of ×2000 and ×4000.

### 2.17. Antimicrobial Activity of Active STMP-Modified Pearl Millet Starch Films Incorporated with FEO

The antimicrobial activity of films was evaluated by the disc diffusion method after seven days of the film elaboration [43]. The bacterial culture was inoculated using an L-spreader, and circular film samples of diameter 20 mm were placed on the solidified agar media surface and incubated at (25 ± 2) ℃ for 5 days. The diameter of the inhibition zone evaluated the antimicrobial activity of the films. All tests were performed in triplicate, and the mean value was observed.

### 2.18. Statistical Analysis

Statistical analyses of the mean of triplicate values data were carried out using MinitabStatistical Software version 14 (Minitab Inc., State College, PA, USA).

## 3. Results and Discussions

### 3.1. Physiochemical Properties of Pearl Millet Starch

#### 3.1.1. Amylose Content

The amylose content of native and sodium trimetaphosphate cross-linked starch is tabulated in Table 1. Native pearl millet starch showed an amylose content of 16.55%. Suma and Urooj [44] reported that amylose content varied from 7.8 to 16% for different pearl millet cultivars. The result showed a significant reduction in the amylose content of the cross-linked starch compared to the native starch. The increase in STMP concentration of 1% to 5% gradually decreased the amylose content of modified starches. The lowest value of amylose content (8.97%) was observed for 5% STMP cross-linked starch. Sharma et al. [17] observed a similar decrease in amylose content for cross-linked starch. The reduction in amylose content of cross-linked starches could be due to intra- and inter-molecular bonding of amylose and amylopectin molecules [45].

#### 3.1.2. Solubility and Swelling Power (SP) of Starch Granules

The solubility and swelling power of the native and STMP cross-linked starches are tabulated in Table 1. The solubility was 12.75% for native starch, while the solubility of STMP cross-linked starches (1%, 3%, and 5%) was observed to be 12.25%, 12.0%, 11.25%, respectively. For native starch, the SP was 15.10 g/g, while swelling power for cross-linked searches (1%, 3%, and 5%) was observed at 14.34, 13.90, and 12.91 g/g, respectively. A significant reduction in the solubility of the starches was observed after modification. It was also observed that both solubility and SP of cross-linked starch reduced with an increase in the levels of cross-linking reagent. Jyothi et al. [46] reported that the addition of cross-linking agents increases the density of starch molecules, resulting in a lower breakdown of starch during gelatinization. This causes the lowering of both solubility and SP of modified starch molecules. Cross-linking modification strengthens the intra- and inter-molecular bonding between the starch chains, restricting starch granules from swelling [3,47].

#### 3.1.3. Light Transmittance

The value of starch paste clarity for native and cross-linked starches was observed for the storage period of 0 to 5 days at 4 °C, and the results are shown in Table 2. The light transmittance value of native pearl millet starch ranged between 0.96% and 2.31%; however, the cross-linked starches values ranged between 1.31 and 4.58%. It was noticed that the values of pasted clarity were decreased with the storage time for native and modified PM starches. There was a gradual decrease in the light transmittance value of the native PM starch, while it was suddenly decreased in cross-linked starches. There was a significant difference (*p* < 0.05) in the transmittance values between native and cross-linked starch. Initially, the light transmittance value was almost doubled after 1% STMP addition. However, it was first decreased then increased slightly with 3% and 5% STMP.

Further, during each storage day, the transmittance values for all cross-linked pearl millet starches were significantly (*p* ≤ 0.05) higher than the corresponding native pearl millet starch. Liu et al. [48] also reported that the retrogradation tendency of cross-linked starch was weaker than the native starch. This might occur due to phosphate groups, which bind with water and form hydrogen bonds with hydroxyl groups of amylose, limiting hydrogen bonds for rearranging and reassociating [49].

### 3.2. Native and Cross-Linked Pearl Millet Starch Films

#### 3.2.1. Moisture Content

The moisture content of the starch film is tabulated in Table 3. Moisture content for the native and modified starch film was observed as 24.16% and 22.44%, respectively. The moisture content of the modified starch film was low compared to native starch film. Gutiérrez et al. [49] also observed decreased moisture content of cross-linked starch film compared with native. This suggests that the reduction in the moisture content of cross-linked films could be due to the addition of the phosphorus group, which enhances the chemical interactions within the films [17]. In the case of oil-added native and cross-linked starch films, moisture content was observed at 20.89% and 19.58%, respectively. The results show that the addition of oil results in low moisture content. Cai et al. [50] also reported an increase in moisture content of starch films on the addition of essential oil. It was concluded that the decrease in moisture content of the oil-added film was due to the reduction in free hydrophilic groups.

#### 3.2.2. Opacity

The opacity of starch films is presented in Table 3. The opacity of native and cross-linked starch film was 0.735% and 0.897%, respectively. A higher transparency value (opacity) represents a lower transmittance of films, i.e., greater opacity. Woggum et al. [51] also reported increased opacity of the cross-linked films compared to native film. They concluded increased opacity was due to the bulk of monostarch phosphate in the cross-linked starch film, resulting in higher transmittance. The opacity of oil-added native and cross-linked starch films was 1.08% and 1.20%. The oil addition results in increasing the thickness of the film, thus leading to an increase in the opaqueness of the film.

#### 3.2.3. Thickness

The observed thickness of the native and cross-linked starch film was 0.18 and 0.20 mm, respectively (Table 3). It was observed that films made with cross-linked starch had more thickness than native starch films. Thickness is considered a vital parameter as it helps determine the mechanical and barrier properties of films. Gutiérrez et al. [52] also reported increased thickness in cross-linked cassava starch films and concluded that cross-linking strengthens the internal structure of starch granules. Additionally, bulky phosphate groups provide starch granules with higher molar volume. It increases the thickness of cross-linked starch films. The thickness of the oil-added native and the cross-linked starch film was 0.20 and 0.22 mm, respectively. The addition of fenugreek oil leads to an increase in the thickness of the films. Sharma et al. [18] also reported the increased thickness of starch films on oil addition. They concluded that it could be due to the essential oil distribution pattern in the film’s matrix.

#### 3.2.4. Water Solubility of Films

The results for the water solubility of starch films are shown in Table 3. The solubility value of the native and cross-linked starch films were 14.77% and 13.15%, respectively. A similar decrease in water solubility for faba bean starch film was observed by Sharma et al. [17]. Garcia et al. [53] explained that the number of free hydroxyl groups in the starch chains decreases with the addition of cross-linking agents, which lessens the affinity of the film matrix to water molecules and finally results in reduced solubility. The solubility of oil-added films was 13.02% and 11.73% in native and cross-linked starch films, respectively. Al-Hashimi et al. [54] also observed the addition of oil on film solubility, owing to an increase in the hydrophobic nature of films due to a decrease in free hydroxyl groups. Therefore, the availability of hydroxyl groups and their interaction with water molecules was reduced and led to less solubility. The addition of oil may have resulted in the formation of amylose–lipid complexes with a hydrophobic hole formed in films, which might have lowered the solubility of films [55].

#### 3.2.5. Water Vapor Permeability (WVP)

WVP values of starch films are shown in Table 4. The WVP of the native and cross-linked starch film was 6.98 and 6.01 g.m/Pa.s.m^2^, respectively. The cross-linked starch films showed superior water barrier properties to native starch films. Reddy and Yang [56] also reported decreased value of WVP in cross-linked starch films. It was concluded that the cross-linking reagent limits water absorption by restricting the mobility of starch chains in the amorphous region which results in less WVP. In contrast, oil-added films which are native and cross-linked have a WVP of 9.54 and 8.76 g.m/Pa.s.m^2^, respectively. A similar increase in WVP of starch films upon oil addition was reported by [54,57]. It was concluded that the obtained high WVP is due to hydrogen and covalent interactions between the amylose and amylopectin chains, which alternatively reduce the ability of hydrogen groups to form a hydrophilic bond with water disrupting the affinity of the film.

#### 3.2.6. Mechanical Properties

##### Tensile Strength (TS)

The tensile strength of films is tabulated in Table 4. TS of the native and cross-linked starch film was 3.44 and 5.85 MPa, respectively. The results showed that cross-linked starch films have higher TS than native starch films. Bruni et al. [58] also reported a similar increase in TS in cross-linked corn starch films compared to native film. They concluded that adding a cross-linking agent provides stronger intermolecular interactions in the starch network. Moreover, cross-linking agents make starch less hydrophilic by reacting with the hydroxyl groups of starch, which might account for the increased network resistance of the films. TS of oil-added native and cross-linked starch was observed as 3.40 and 4.59 MPa, respectively. As can be seen, due to the addition of oil, TS of starch films was found to decrease. Souza et al. [22] reported a reduction in the TS upon adding clove essential oil in cassava starch film. It was concluded that the oil addition reduces intermolecular interaction among starch chains, causing lower tensile strength of films.

##### Elongation at Break (EB)

The results for EB of films are shown in Table 4. EB of native and cross-linked starch films was 56.5% and 51.5%, respectively. The results showed that the EB of the native film was higher than cross-linked starch films. Sukhija et al. [40] observed a higher EB in native lotus rhizome starch films than cross-linked starch films. They concluded that the addition of STMP reduces chain mobility by introducing bulky phosphate groups between starch molecules that provide rigidity to films, and hence the EB was reduced. For the films formulated with the addition of oil, the observed EB for the native and cross-linked starch film was 70.2% and 61.5%, respectively. Results showed that the addition of oil to films increased EB. Li et al. [57] also reported the increasing value of EB in sweet potato starch with oregano essential oil films. They concluded that adding oil replaces the stronger intermolecular polysaccharide interactions with weaker polysaccharide–oil interactions, creating more flexible areas within the films.

#### 3.2.7. Thermal Properties

Thermal properties of films were measured using a differential scanning calorimeter (DSC). The resulting thermograms and values are shown in Table 5. Thermal properties studied were transition temperatures—onset temperature (T_o_), peak temperature (T_p_), conclusion temperature (T_c_), and melting enthalpy (ΔH_m_). Native starch films showed T_o_, T_p_, T_c_, and ΔH_m_ of 165.99, 168.39, 172.04°C, and 328.34 J/g, respectively. CL starch films showed T_o_, T_p_, T_c_, and ΔH_m_ of 169.84, 172.63, 178.34°C, and 358.20 J/g, respectively. The CL starch film showed significantly (*p* ≤ 0.05) increased T_p_ and ΔH_m_. A similar increase in T_p_ and ΔH_m_ in cross-linked corn starch films was also observed by Xu et al. [59]. It was concluded that cross-linking results in higher decomposition temperature due to the formation of a compact and stronger network of intra- and inter-molecular bonds on the addition of cross-linking agents. In oil-added films, the T_o_, T_p_, T_c_, and ΔH_m_ values for the native starch film were 167.84, 184.99, 197.39°C, and 468.64 J/g, respectively. For a cross-linked starch film with oil addition, values of T_o_, T_p_, T_c_, and ΔH_m_ were 179.52, 185.68, 197.47 °C, and 498.38 J/g, respectively. The results showed that adding fenugreek oil to films increased T_o_, T_p_, and T_c_ and majorly increased ΔH_m_ for both native and CL starch films. Among oil-added films, cross-linked starch with oil (cross-linked+oil) films had higher T_o_, T_p_, T_c_, and ΔH_m_ values than native starch with oil (N+oil) films. Suput et al. [60] also reported increased thermal temperatures and melting enthalpy upon adding black cumin oil and oregano oil to the corn starch films. They concluded that adding oils to the starch film has a similar effect to a plasticizer, i.e., it increases T_o_, T_p_, T_c_, and ΔH_m_ of films by facilitating chain mobility.

#### 3.2.8. Morphological Properties

**Figure 1:**
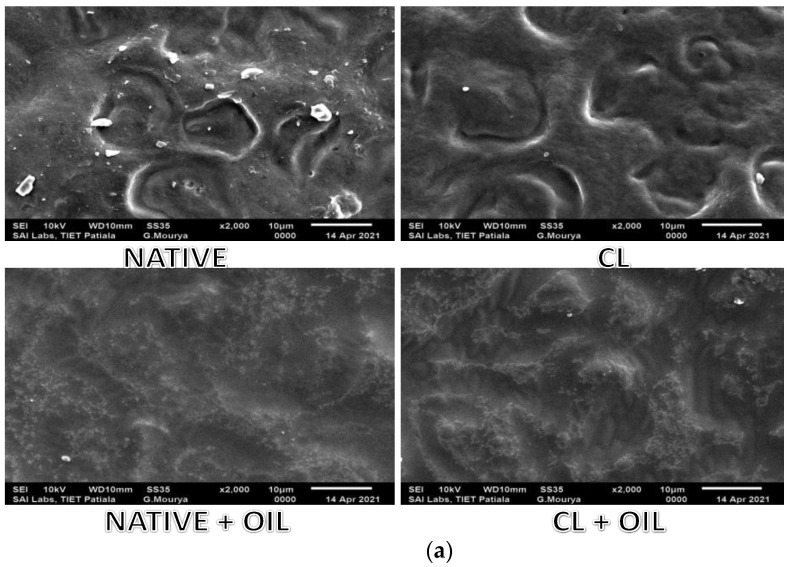

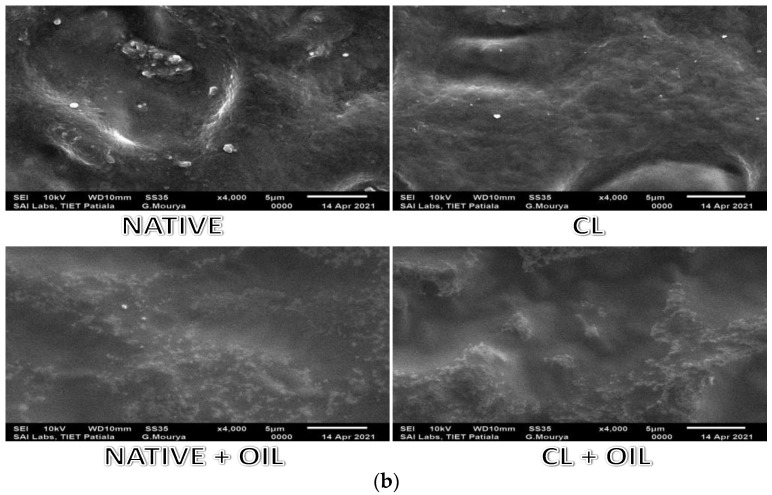
Scanning electron micrographs (SEM) of native and cross-linked starch and films loaded with fenugreek oil (**a**) at ×2000. (**b**) at ×4000.

The scanning electron microscopy (SEM) images of the surfaces of pearl millet starch films are shown in Figure 1a,b when viewed under magnification of 2000X and 4000X, respectively, at 10 kv. It was seen that the native film possessed the roughest structure, with many cracks, pores, and ridges. On the other hand, cross-linked starch films showed improved morphological characteristics, with a smooth surface free from cracks, pores, ridges, or breaks. Liang et al. [61] also observed improved surface smoothness in cross-linked chitosan/bacterial cellulose composite film. It was concluded that a cross-linking reagent provides compactness and uniformity to the film structure, indicating that cross-linkers create closer interaction between matrix molecules. However, in oil-added films, it was observed that adding oil provides a smoother surface than native and cross-linked starch films without oil addition.

#### 3.2.9. Antibacterial Activity of Active Pearl Millet Starch Films

The inhibition areas showed by native and modified starch film disks with and without fenugreek essential oil against studied bacteria are shown in Table 6. The native starch film showed no inhibition zone, whereas film incorporated with fenugreek oil showed 40.22% and 41.53% inhibition areas, respectively. It was observed that cross-linked starch films without oil addition showed a very little inhibition zone of 1.34%. Table 6 shows the inhibition of fenugreek oil-loaded films against *E*. *coli*. The results proved that even at a minimum concentration, fenugreek oil would be an effective antimicrobial agent without altering the sensory characteristics of packaged food. Sulieman et al. [62] also reported a good inhibitory effect of fenugreek oil against *E. coli*. Limited studies have been conducted on the antimicrobial characteristics of starch and essential oils films. Nevertheless, no information has been presented about the effect of fenugreek oil on the addition of starch films.

The present study contributes to ongoing research on oils derived from plants (leaves, buds, fruits, flowers, herbs, seeds, etc.) with potential antimicrobial properties against some common fungi. Their incorporation into different films (such as edible starch films) has been gaining increasing attention within the food industry and other industries [60,63,64,65,66,67]. Fenugreek seed oil has been intensively studied due to its medicinal use. Still, its use as edible oil, food stabilizer, and emulsifying agent, or further applications such as film/coatings, has not been explored to date [68,69].

## 4. Conclusions

Cross-linking of starch and oil incorporation significantly affected the physicochemical, thermal, mechanical, morphological, and antimicrobial characteristics of pearl millet starch films. Significant differences were observed for moisture, thickness, opacity, water-solubility, mechanical, thermal, and antibacterial characteristics of films. Water vapor permeability rate(WVPR) was decreased in cross-linked (CL) starch films and increased after oil addition. The tensile strength of CL starch film was highest, while a native film with added oil showed the highest value for elongation break. The CL starch film showed higher thermal transition temperatures (To, Tp, Tc) and melting enthalpy (ΔHm), indicating that modified starch films have a more compact internal structure. CL starch also enhanced the morphological properties of films to a greater extent, resulting in a compact, uniform, and homogeneous structures with improved surface smoothness. This indicated a closer interaction of matrix molecules induced by cross-linkers. Oil-added films showed an inhibition zone against *E. coli* compared to native and CL starch films without oil, confirming the inhibitory effect of fenugreek oil, even at low concentrations. In summary, CL starch improved the barrier and mechanical properties, while fenugreek oil addition enhanced the antimicrobial potential of films. This suggests different applications, such as flexible packaging of food products and as a good preservation material to maintain quality attributes and extend their shelf-life. Further, being an underutilized crop, pearl millet can be utilized by starch-based industries as a good alternative for conventional cereal starches.

## Figures and Tables

**Table 1:** Amylose content, swelling power, and solubility of native and cross-linked starches.

Pearl Millet Starch	Amylose Content (%)	SwellingPower (g/g)	Solubility (%)
Native	16.55 ± 0.21 ^c^	15.10 ± 0.23 ^d^	12.75 ± 0.20 ^c^
CL—1%	13.31 ± 0.20 ^b^	14.34 ± 0.21 ^c^	12.25 ± 0.19 ^b^
CL—3%	13.23 ± 0.32 ^b^	13.90 ± 0.30 ^b^	12.00 ± 0.21 ^b^
CL—5%	8.97 ± 0.22 ^a^	12.91 ± 0.21 ^a^	11.25 ± 0.17 ^a^

Means ± Standard Deviation (*n* = 3) of triplicate analysis. Means followed by the same letter within the column do not differ significantly (*p* < 0.05). CL—1% (cross-linking with 1% Sodium trimetaphosphate (*STMP*) CL—3% (cross-linking with 3% Sodium trimetaphosphate (*STMP*), CL—5% (cross-linking with 5% Sodium trimetaphosphate (*STMP*)).

**Table 2:** Light transmittance of native and cross-linked pearl millet starches.

Pearl Millet Starch	Day 0	Day 1	Day 2	Day 3	Day 4	Day 5
Native	2.31 ± 0.02 ^a^	1.61 ± 0.01 ^a^	1.24 ± 0.02 ^a^	1.12 ± 0.01 ^a^	1.04 ± 0.02 ^a^	0.96 ± 0.03 ^a^
CL—1%	4.58 ± 0.01 ^d^	2.46 ± 0.02 ^c^	1.74 ± 0.01 ^b^	1.57 ± 0.02 ^b^	1.35 ± 0.01 ^b^	1.31 ± 0.01 ^b^
CL—3%	4.37 ± 0.01 ^b^	2.29 ± 0.03 ^b^	1.76 ± 0.02 ^b^	1.58 ± 0.01 ^b^	1.42 ± 0.03 ^c^	1.35 ± 0.02 ^c^
CL—5%	4.43 ± 0.02 ^c^	2.60 ± 0.01 ^d^	1.78 ± 0.01 ^b^	1.58 ± 0.03 ^b^	1.45 ± 0.01 ^c^	1.35 ± 0.02 ^c^

Means ± Standard Deviation (*n* = 3) of triplicate analysis. Means followed by the same letter within the column do not differ significantly (*p* < 0.05). CL—1% (cross-linking with 1% Sodium trimetaphosphate (*STMP*) CL—3% (cross-linking with 3% Sodium trimetaphosphate (*STMP*), CL—5% (cross-linking with 5% Sodium trimetaphosphate (*STMP*)).

**Table 3:** Moisture content, thickness, opacity, and water solubility of native and cross-linked starch and films loaded with fenugreek oil films.

Films	Moisture Content (%)	Thickness (mm)	Opacity (%)	Water Solubility (%)
Native	24.16 ± 0.22 ^d^	0.18 ± 0.001 ^a^	0.735 ± 0.04 ^a^	14.77 ± 0.16 ^c^
Cross-linked	22.44 ± 0.21 ^c^	0.20 ± 0.002 ^b^	0.897 ± 0.03 ^b^	13.15 ± 0.19 ^b^
Native+oil	20.89 ± 0.19 ^b^	0.20 ± 0.003 ^b^	1.08 ± 0.03 ^c^	13.02 ± 0.18 ^b^
Cross-linked+oil	19.58 ± 0.20 ^a^	0.22 ± 0.001 ^c^	1.20 ± 0.02 ^d^	11.73 ± 0.23 ^a^

Means ± Standard Deviation (*n* = 3) of triplicate analysis. Means followed by the same letter within the column do not differ significantly (*p* < 0.05). Native (films prepared from native pearl millet starch), Native+oil (films prepared from native pearl millet starch loaded with fenugreek oil), Cross-linked (films prepared from cross-linked pearl millet starch), Cross linked+oil (films prepared from cross-linked pearl millet starch loaded with fenugreek oil).

**Table 4:** Mechanical properties and water vapor permeability (WVP) of native and cross-linked starch and films loaded with fenugreek oil films.

Films	Tensile Strength (MPa)	Elongation at Break (%)	WVP (g.m/Pa.s.m^2^)
Native	3.44 ± 0.35 ^a^	56.5 ± 2.35 ^b^	6.98 ± 0.003 ^b^
Cross-linked	5.85 ± 0.38 ^c^	51.5 ± 2.12 ^a^	6.01 ± 0.004 ^a^
Native+oil	3.40 ± 0.42 ^a^	70.2 ± 1.74 ^d^	9.54 ± 0.002 ^d^
Cross-linked +oil	4.59 ± 0.45 ^b^	61.5 ± 1.25 ^c^	8.76 ± 0.002 ^c^

Means ± Standard Deviation (*n* = 3) of triplicate analysis. Means followed by the same letter within the column do not differ significantly (*p* < 0.05). Native (films prepared from native pearl millet starch), Native+oil (films prepared from native pearl millet starch loaded with fenugreek oil), Cross-linked (films prepared from cross-linked pearl millet starch), Cross linked+oil (films prepared from cross-linked pearl millet starch loaded with fenugreek oil).

**Table 5:** Thermal properties of native and cross-linked starch and films loaded with fenugreek oil.

Films	T_o_ (°C)	T_p_ (°C)	T_c_ (°C)	ΔH_m_ (J/g)
Native	165.99 ± 3.3 ^a^	168.39 ± 6 ^a^	172.04 ± 4 ^a^	328.34 ± 5 ^a^
Cross-linked	169.84 ± 4.8 ^c^	172.63 ± 5 ^b^	178.34 ± 6 ^b^	358.20 ± 8 ^b^
Native+oil	167.84 ± 5.4 ^b^	184.99 ± 4 ^c^	197.39 ± 3 ^c^	468.64 ± 4 ^c^
Cross-linked+oil	179.52 ± 3.8 ^d^	185.68 ± 7 ^d^	197.47 ± 5 ^c^	498.38 ± 6 ^d^

Means ± Standard Deviation (*n* = 3) of triplicate analysis. Means followed by the same letter within the column do not differ significantly (*p* < 0.05). Native (films prepared from native pearl millet starch), Native+oil (films prepared from native pearl millet starch loaded with fenugreek oil), Cross-linked (films prepared from cross-linked pearl millet starch), Cross linked+oil (films prepared from cross-linked pearl millet starch loaded with fenugreek oil). T_o_ = Onset temperature, T_p_ = Peak temperature, T_c_ = Conclusion temperature, ΔH_m_ = melting enthalpy (dwb, based on starch weight), N (native), CL (cross-linked).

**Table 6:** Inhibition area (%) against *E*. ***c****oli* of native and cross-linked starch and films loaded with fenugreek oil.

Films	Inhibition Area %
Native	0.00 ± 0.00 ^a^
Cross-linked	1.34 ± 0.58 ^b^
Native+oil	40.22 ± 3.48 ^c^
Cross-linked+oil	41.53 ± 2.83 ^c^

Means ± Standard Deviation (*n* = 3) of triplicate analysis. Means followed by the same letter within the column do not differ significantly (*p* < 0.05). Native (films prepared from native pearl millet starch), Cross-linked (films prepared from cross-linked pearl millet starch), Native+oil (films prepared from native pearl millet starch loaded with fenugreek oil), Cross linked+oil (films prepared from cross-linked pearl millet starch loaded with fenugreek oil).

## Data Availability

Not applicable.

## References

[1] Hong M., Chen E.Y.-X. (2017). Chemically recyclable polymers: A circular economy approach to sustainability. Green Chem..

[2] Chen G., Feng Q., Wang J. (2020). Mini-review of microplastics in the atmosphere and their risks to humans. Sci. Total. Environ..

[3] Bangar S.P., Purewal S.S., Trif M., Maqsood S., Kumar M., Manjunatha V., Rusu A.V. (2021). Functionality and Applicability of Starch-Based Films: An Eco-Friendly Approach. Foods.

[4] Jiang T., Duan Q., Zhu J., Liu H., Yu L. (2020). Starch-based biodegradable materials: Challenges and opportunities. Adv. Ind. Eng. Polym. Res..

[5] Żołek-Tryznowska Z., Kałuża A. (2021). The Influence of Starch Origin on the Properties of Starch Films: Packaging Performance. Materials.

[6] Sharma M., Yadav D.N., Singh A.K., Tomar S.K. (2015). Rheological and functional properties of heat moisture treated pearl millet starch. J. Food Sci. Technol..

[7] Sandhu K.S., Siroha A.K. (2017). Relationships between physicochemical, thermal, rheological and in vitro digestibility properties of starches from pearl millet cultivars. LWT-Food Sci. Technol..

[8] Shaikh M., Ali T.M., Hasnain A. (2015). Post succinylation effects on morphological, functional and textural characteristics of acid-thinned pearl millet starches. J. Cereal Sci..

[9] Shaikh M., Haider S., Ali T.M., Hasnain A. (2019). Physical, thermal, mechanical and barrier properties of pearl millet starch films as affected by levels of acetylation and hydroxypropylation. Int. J. Biol. Macromol..

[10] Punia S., Kumar M., Siroha A.K., Kennedy J.F., Dhull S.B., Whiteside W.S. (2021). Pearl millet grain as an emerging source of starch: A review on its structure, physicochemical properties, functionalization, and industrial applications. Carbohydr. Polym..

[11] Kocira A., Kozłowicz K., Panasiewicz K., Staniak M., Szpunar-Krok E., Hortyńska P. (2021). Polysaccharides as Edible Films and Coatings: Characteristics and Influence on Fruit and Vegetable Quality—A Review. Agronomy.

[12] Siroha A.K., Bangar S.P., Sandhu K.S., Trif M., Kumar M., Guleria P. (2021). Effect of Cross-Linking Modification on Structural and Film-Forming Characteristics of Pearl Millet (*Pennisetum glaucum* L.) Starch. Coatings.

[13] Lauer M.K., Smith R.C. (2020). Recent advances in starch-based films toward food packaging applications: Physicochemical, mechanical, and functional properties. Compr. Rev. Food Sci. Food Saf..

[14] Colivet J., Carvalho R. (2017). Hydrophilicity and physicochemical properties of chemically modified cassava starch films. Ind. Crop. Prod..

[15] Nawaz H., Waheed R., Nawaz M., Shahwar D. (2020). Physical and Chemical Modifications in Starch Structure and Reactivity. Chem. Prop. Starch.

[16] Bangar S.P., Sandhu K.S., Rusu A.V., Kaur P., Purewal S.S., Kaur M., Kaur N., Trif M. (2021). Proso-Millet-Starch-Based Edible Films: An Innovative Approach for Food Industries. Coatings.

[17] Sharma V., Kaur M., Sandhu K.S., Godara S.K. (2020). Effect of cross-linking on physico-chemical, thermal, pasting, in vitro digestibility and film forming properties of Faba bean (*Vicia faba* L.) starch. Int. J. Biol. Macromol..

[18] Sharma S., Barkauskaite S., Jaiswal A.K., Jaiswal S. (2021). Essential oils as additives in active food packaging. Food Chem..

[19] Anand S.P., Sati N. (2013). Artificial preservatives and their harmful effects: Looking toward nature for safer alternatives. Int. J. Pharm.Sci. Res..

[20] Ciocan-Cartita C.A., Jurj A., Zanoaga O., Ciocan-Cârtiţăa C.A., Cojocneanua R., Moldovana C., Radulya L., Pop-Bicaa C., Trifb M., Irimiec A. (2020). New insights in gene expression alteration as effect of doxorubicin drug resistance in triple negative breast cancer cells. J. Exp. Cancer Res..

[21] Carpena M., Nuñez-Estevez B., Soria-Lopez A., Garcia-Oliveira P., Prieto M.A. (2021). Essential Oils and Their Application on Active Packaging Systems: A Review. Resources.

[22] Souza A., Goto G., Mainardi J., Coelho A., Tadini C. (2013). Cassava starch composite films incorporated with cinnamon essential oil: Antimicrobial activity, microstructure, mechanical and barrier properties. LWT.

[23] Szabo K., Dulf F.V., Teleky B.-E., Eleni P., Boukouvalas C., Krokida M., Kapsalis N., Rusu A.V., Socol C.T., Vodnar D.C. (2021). Evaluation of the Bioactive Compounds Found in Tomato Seed Oil and Tomato Peels Influenced by Industrial Heat Treatments. Foods.

[24] Alwhibi M.S., Soliman D.A. (2014). Evaluating the antibacterial activity of fenugreek (Trigonella foenum-graecum) seed extract against a selection of different pathogenic bacteria. J. Pure Appl. Microbiol..

[25] Nandagopal S., Dhanalakshmi D.P., Kumar A.G., Sujitha D.J.J.P.R. (2012). Phytochemical and antibacterial studies of fenugreek Trigonella foenum-graecum L.—A multipurpose medicinal plant. J. Pharm. Res..

[26] Siroha A.K., Punia S. (2020). Starch: Structure, Properties and Applications. Pearl Millet.

[27] Woo K.S., Seib P.A. (2002). Cross-Linked Resistant Starch: Preparation and Properties. Cereal Chem. J..

[28] Akbari S., Abdurahman N.H., Yunus R.M., Alara O.R., Abayomi O.O. (2019). Extraction, characterization and antioxidant activity of fenugreek (Trigonella-Foenum Graecum) seed oil. Mater. Sci. Energy Technol..

[29] Trif M., Socaciu C. (2008). Evaluation of effiency, release and oxidation stability of seabuckthorn microencapsulated oil using Fourier transformed infrared spectroscopy. Chem. Listy.

[30] Williams P.C., Kuzina F.D., Hlynka I. (1970). Rapid colorimetric procedure for estimating the amylose content of starches and flours. Cereal Chem..

[31] Patil P.D., Gokhale M.V., Chavan N.S. (2014). Mango starch: Its use and future prospects. Innov. J. Food Sci..

[32] Leach H.W. (1959). Structure of starch granules. I. Swelling and solubility patterns of various starches. Cereal Chem..

[33] Wulff D., Chan A., Liu Q., Gu F.X., Aucoin M.G. (2020). Characterizing internal cavity modulation of corn starch microcapsules. Heliyon.

[34] Kumar R., Khatkar B.S. (2017). Thermal, pasting and morphological properties of starch granules of wheat (*Triticum aestivum* L.) varieties. J. Food Sci. Technol..

[35] Perera C., Hoover R. (1999). Influence of hydroxypropylation on retrogradation properties of native, defatted and heat-moisture treated potato starches. Food Chem..

[36] Rusu A.V., Criste F.L., Mierliţă D., Socol C.T., Trif M. (2020). Formulation of Lipoprotein Microencapsulated Beadlets by Ionic Complexes in Algae-Based Carbohydrates. Coatings.

[37] Ramos Ó.L., Reinas I., Silva S.I., Fernandes J.C., Cerqueira M.A., Pereira R.N., Malcata F.X. (2013). Effect of whey protein purity and glycerol content upon physical properties of edible films manufactured therefrom. Food Hydrocoll..

[38] Romero-Bastida C.A., Bello-Pérez L.A., García M.A., Martino M.N., Solorza-Feria J., Zaritzky N.E. (2005). Physicochemical and microstructural characterization of films prepared by thermal and cold gelatinization from non-conventional sources of starches. Carbohydr. Polym..

[39] (2015). ASTM E96/E96M-15. Standard Test Methods for Water Vapor Transmission of Materials. Annual Book of ASTM Standards.

[40] Sukhija S., Singh S., Riar C.S. (2019). Development and characterization of biodegradable films from whey protein concentrate, psyllium husk and oxidized, crosslinked, dual-modified lotus rhizome starch composite. J. Sci. Food Agric..

[41] (2012). ASTM D882-12, Standard test method for tensile properties of thin plastic sheeting. Annual Book of ASTM Standards.

[42] Sandhu K.S., Sharma L., Kaur M., Kaur R. (2020). Physical, structural and thermal properties of composite edible films prepared from pearl millet starch and carrageenan gum: Process optimization using response surface methodology. Int. J. Biol. Macromol..

[43] Farooq M., Azadfar E., Rusu A., Trif M., Poushi M.K., Wang Y. (2021). Improving the Shelf Life of Peeled Fresh Almond Kernels by Edible Coating with Mastic Gum. Coatings.

[44] Suma P.F., Urooj A. (2015). Isolation and characterization of starch from pearl millet (*Pennisetum typhoidium*) flours. Int. J. Food Prop..

[45] Hazarika B.J., Sit N. (2016). Effect of dual modification with hydroxypropylation and cross-linking on physicochemical properties of taro starch. Carbohydr. Polym..

[46] Jyothi A.N., Moorthy S.N., Rajasekharan K.N. (2006). Effect of Cross-linking with Epichlorohydrin on the Properties of Cassava (Manihot esculenta Crantz) Starch. Starch/Stärke.

[47] Singh J., Kaur L., McCarthy O.J. (2007). Factors influencing the physico-chemical, morphological, thermal and rheological properties of some chemically modified starches for food applications—A review. Food Hydrocoll..

[48] Liu J., Wang B., Lin L., Zhang J., Liu W., Xie J., Ding Y. (2014). Functional, physicochemical properties and structure of cross-linked oxidized maize starch. Food Hydrocoll..

[49] Gutiérrez T.J., Morales N.J., Pérez E., Tapia M.S., Famá L. (2015). Physico-chemical properties of edible films derived from native and phosphatedcush-cush yam and cassava starches. Food Packag. Shelf Life.

[50] Cai C., Ma R., Duan M., Deng Y., Liu T., Lu D. (2020). Effect of starch film containing thyme essential oil microcapsules on physicochemical activity of mango. LWT.

[51] Woggum T., Sirivongpaisal P., Wittaya T. (2014). Properties and characteristics of dual-modified rice starch based biodegradable films. Int. J. Biol. Macromol..

[52] Gutiérrez T., Tapia M.S., Pérez E., Famá L. (2015). Structural and mechanical properties of edible films made from native and modified cush-cush yam and cassava starch. Food Hydrocoll..

[53] Garcia P.S., Grossmann M.V.E., Shirai M.A., Lazaretti M.M., Yamashita F., Muller C.M.O., Mali S. (2014). Improving action of citric acid as compatibiliser in starch/polyester blown films. Ind. Crop. Prod..

[54] G Al-Hashimi A., Ammar A.B., Cacciola F., Lakhssassi N. (2020). Development of a Millet Starch Edible Film Containing Clove Essential Oil. Foods.

[55] López C.A., de Vries A.H., Marrink S. (2012). Amylose folding under the influence of lipids. Carbohydr. Res..

[56] Reddy N., Yang Y. (2010). Citric acid cross-linking of starch films. Food Chem..

[57] Li J., Ye F., Lei L., Zhao G. (2018). Combined effects of octenylsuccination and oregano essential oil on sweet potato starch films with an emphasis on water resistance. Int. J. Biol. Macromol..

[58] Bruni G.P., de Oliveira J.P., El Halal S.L.M., Flores W.H., Gundel A., de Miranda M.Z., da Rosa Zavareze E. (2018). Phosphorylated and cross-linked wheat starches in the presence of polyethylene oxide and their application in biocomposite films. Starch-Stärke.

[59] Xu H., Canisag H., Mu B., Yang Y. (2015). Robust and Flexible Films from 100% Starch Cross-Linked by Biobased Disaccharide Derivative. ACS Sustain. Chem. Eng..

[60] Šuput D., Lazić V., Pezo L., Markov S., Vaštag Ž., Popović L., Radulović A., Ostojic S., Zlatanovic S., Popović S. (2016). Characterization of Starch Edible Films with Different Essential Oils Addition. Pol. J. Food Nutr. Sci..

[61] Liang J., Wang R., Chen R. (2019). The Impact of Cross-linking Mode on the Physical and Antimicrobial Properties of a Chitosan/Bacterial Cellulose Composite. Polymers.

[62] Sulieman A.M.E., Ahmed H.E., Abdelrahim A.M. (2008). The chemical composition of fenugreek (*Trigonella foenumgraceum* L.) and the antimicrobial properties of its seed oil. Gezira J. Eng. Appl. Sci..

[63] Dietrich T., Velasco M.V., Echeverría P., Pop B., Rusu A. (2016). Crop and Plant Biomass as Valuable Material for BBB. Alternatives for Valorization of Green Wastes. Biotransformation of Agricultural Waste and By-Products.

[64] Swamy M.K., Akhtar M.S., Sinniah U.R. (2016). Antimicrobial properties of plant essential oils against human pathogens and their mode of action: An updated review. Evidence-Based Complement. Altern. Med..

[65] Mitrea L., Ranga F., Fetea F., Dulf F.V., Rusu A., Trif M., Vodnar D.C. (2019). Biodiesel-Derived Glycerol Obtained from Renewable Biomass-A Suitable Substrate for the Growth of Candida zeylanoides Yeast Strain ATCC 20367. Microorganisms.

[66] Figueroa-Lopez K.J., Vicente A.A., Reis M.A.M., Torres-Giner S., Lagaron J.M. (2019). Antimicrobial and Antioxidant Performance of Various Essential Oils and Natural Extracts and Their Incorporation into Biowaste Derived Poly(3-hydroxybutyrate-co-3-hydroxyvalerate) Layers Made from Electrospun Ultrathin Fibers. Nanomaterials.

[67] Chouhan S., Sharma K., Guleria S. (2017). Antimicrobial Activity of Some Essential Oils—Present Status and Future Perspectives. Medicines.

[68] Rekik D.M., Khedir S.B., Moalla K.K., Kammoun N.G., Rebai T., Sahnoun Z. (2016). Evaluation of wound healing properties of grapeseed, sesame, and fenugreek oils. Evid. Based Complement. Altern. Med..

[69] Munshi M., Arya P., Kumar P. (2020). Physico-Chemical Analysis and Fatty Acid Profiling of Fenugreek (Trigonella foenum-graecum) Seed Oil Using Different Solvents. J. Oleo Sci..

